# Supplementary figures and images for: A food color-based colorimetric assay for Cryptococcus neoformans laccase activity
Source: Microbiol Spectr. 2024 Jun 13;12(8):e00442-24. doi: 10.1128/spectrum.00442-24 (PMC11302723; doi:10.1128/spectrum.00442-24)

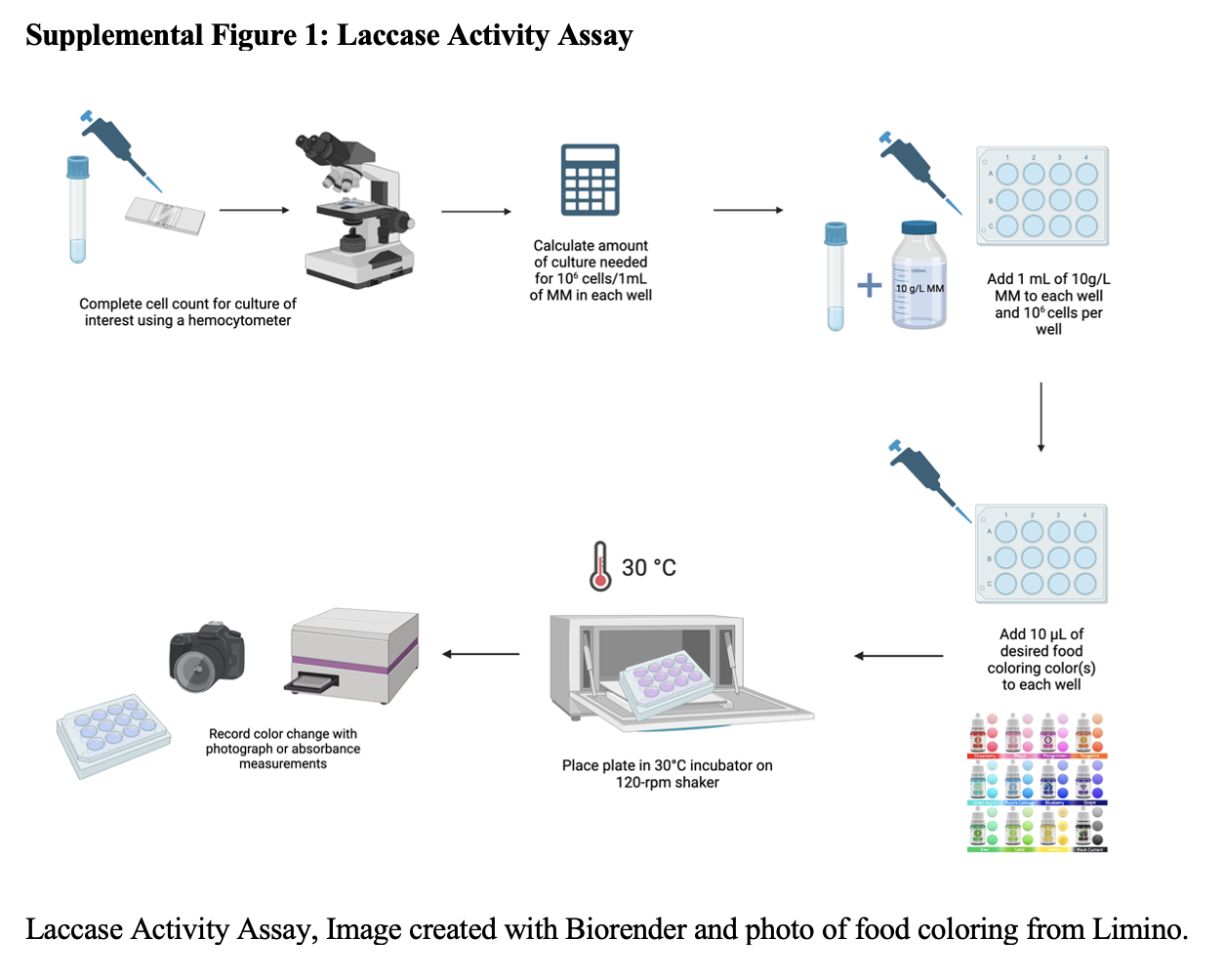

Supplement: Figure S1 — Colorimetric Assay Methods Flowchart. [file spectrum.00442-24-s0001.tiff]

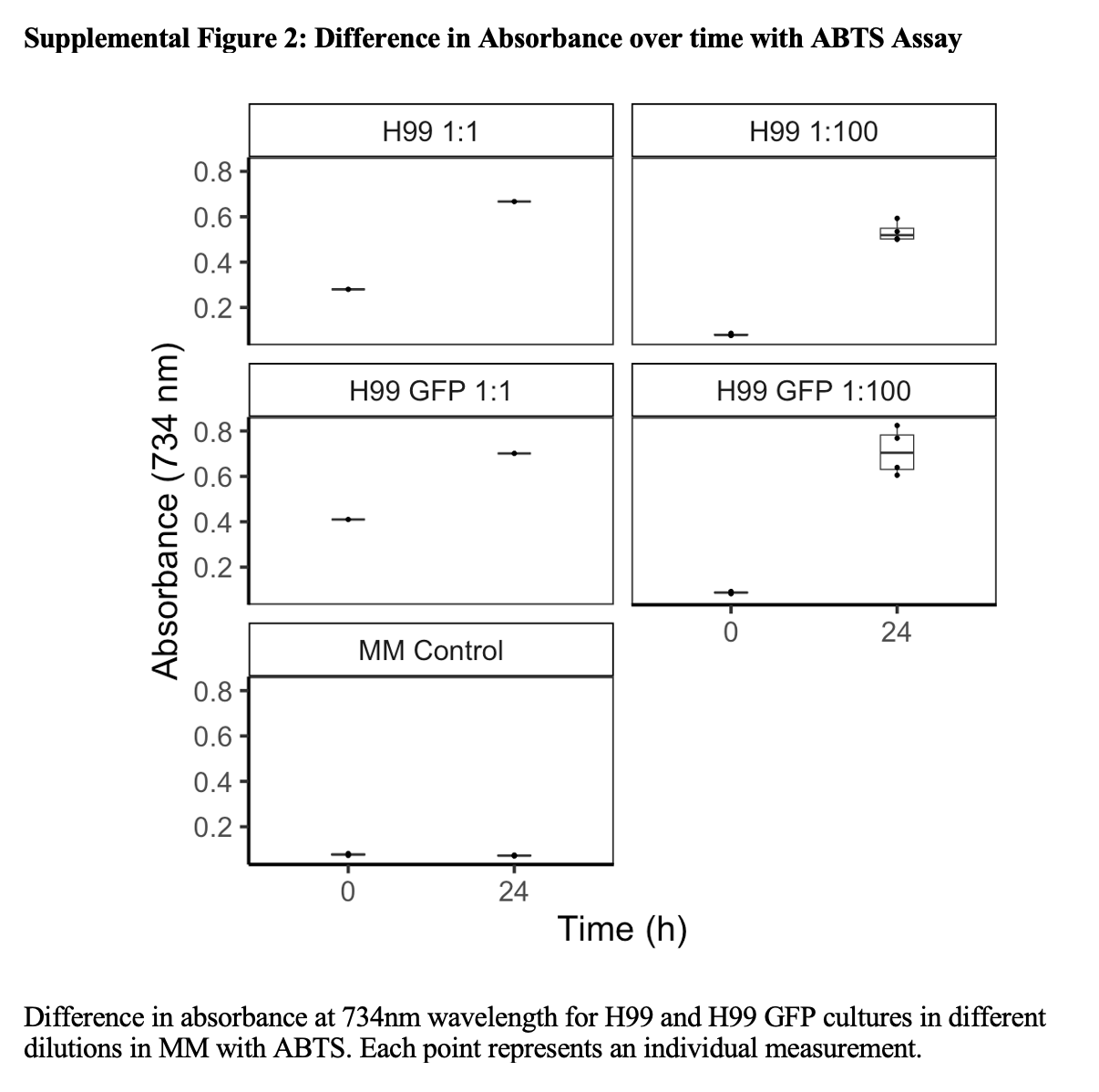

Supplement: Figure S2 — ABTS Assay Absorbance Measurements. [file spectrum.00442-24-s0002.tiff]
