# Supplementary material for: A food color-based colorimetric assay for Cryptococcus neoformans laccase activity
Source: Microbiol Spectr. 2024 Jun 13;12(8):e00442-24. doi: 10.1128/spectrum.00442-24 (PMC11302723; doi:10.1128/spectrum.00442-24)
Supplement: Table S1 — Limino Food Coloring Ingredients. [file spectrum.00442-24-s0003.docx]

**Supplemental Table 1: Limino Food Coloring Ingredients**

| **Color Name** | **Food Coloring Components** | **CAS No.** |
| --- | --- | --- |
| Blueberry | Acid Red 27 | 915-67-3 |
|  | Food Blue No. 1 | 3844-45-9 |
| Lemon | Food Yellow No. 4 | 1934-21-0 |
|  | Food Yellow 3 | 2783-94-0 |
| Lime | Food Yellow No. 4 | 1934-21-0 |
|  | Food Blue No. 1 | 3844-45-9 |
| Mangosteen | Acid Red 27 | 915-67-3 |
|  | Food Blue No. 1 | 3844-45-9 |
| Purple Cabbage | Food Blue No. 1 | 3844-45-9 |
|  | Food Yellow 3 | 2783-94-0 |
|  | Acid Red 27 | 915-67-3 |
| Strawberry | Food Red 7 | 2611-82-7 |
|  | Food Yellow 3 | 2783-94-0 |
| Tangerine | Food Yellow 3 | 2783-94-0 |
|  | Food Red 7 | 2611-82-7 |

Ingredients added to the mixture by the manufacturer (amounts proprietary to Limino): Sorbitol (CAS No. 50-70-4), Water (CAS No. 7732-18-5), Glycerin (CAS No. 56-81-5), Carboxymethylcellulose sodium (CAS No. 9004-32-4), Potassium sorbate (CAS No. 590-00-1)
